# Occurrence and phylogenetic analysis of bovine respiratory syncytial virus in outbreaks of respiratory disease in Norway

**DOI:** 10.1186/1746-6148-10-15

**Published:** 2014-01-14

**Authors:** Thea B Klem, Espen Rimstad, Maria Stokstad

**Affiliations:** 1 Department of Production Animal Clinical Sciences, Norwegian University of Life Sciences, P.O. Box 8146 Dep., Oslo N-0033, Norway; 2 Department of Food Safety & Infection Biology, Norwegian University of Life Sciences, P.O. Box 8146 Dep., Oslo N-0033, Norway

**Keywords:** Bovine respiratory syncytial virus, Real time PCR, Occurrence, Phylogenetic analysis

## Abstract

**Background:**

Bovine respiratory syncytial virus (BRSV) is one of the major pathogens involved in the bovine respiratory disease (BRD) complex. The seroprevalence to BRSV in Norwegian cattle herds is high, but its role in epidemics of respiratory disease is unclear. The aims of the study were to investigate the etiological role of BRSV and other respiratory viruses in epidemics of BRD and to perform phylogenetic analysis of Norwegian BRSV strains.

**Results:**

BRSV infection was detected either serologically and/or virologically in 18 (86%) of 21 outbreaks and in most cases as a single viral agent. When serology indicated that bovine coronavirus and/or bovine parainfluenza virus 3 were present, the number of BRSV positive animals in the herd was always higher, supporting the view of BRSV as the main pathogen. Sequencing of the G gene of BRSV positive samples showed that the current circulating Norwegian BRSVs belong to genetic subgroup II, along with other North European isolates. One isolate from an outbreak in Norway in 1976 was also investigated. This strain formed a separate branch in subgroup II, clearly different from the current Scandinavian sequences. The currently circulating BRSV could be divided into two different strains that were present in the same geographical area at the same time. The sequence variations between the two strains were in an antigenic important part of the G protein.

**Conclusion:**

The results demonstrated that BRSV is the most important etiological agent of epidemics of BRD in Norway and that it often acts as the only viral agent. The phylogenetic analysis of the Norwegian strains of BRSV and several previously published isolates supported the theory of geographical and temporal clustering of BRSV.

## Background

Bovine respiratory disease (BRD) is of significant importance for production and animal welfare in the cattle industry [1-3]. The bovine respiratory syncytial virus (BRSV) is one of the major contributors to BRD, inducing clinical signs that may vary from none to fatal [4,5]. The seroprevalence is generally found to be high, with up to 100% seropositive herds in some areas [6-10]. A recent nationwide serological study indicated that 54% of the dairy herds in Norway had BRSV present in the herd the previous year [11]. BRSV infections can appear as epidemics with outbreaks affecting many animals in several herds within the same area or as part of protracted respiratory disease problems in single herds, i.e. enzootic pneumonia. BRD epidemics occur frequently in Norway during the winter, when the animals are housed indoors. BRSV was isolated in Norway already in 1976 during a severe epizootic outbreak which spread throughout most of the country [12]. Only sporadic outbreaks were reported in the following years, but in 1995 another severe epidemic was reported [13]. The Norwegian cattle population is currently free from bovine herpes virus type 1 (BHV 1) [14] and bovine virus diarrhoea virus (BVDV) [15], and *Mycoplasma bovis* has never been detected [16].

BRSV belongs to the genus *Pneumovirus* in the family *Paramyxoviridae*. The gene coding for the surface cell attachment glycoprotein (G protein) has the highest reported mutation rate and is commonly used as the target for investigations involving molecular epidemiology and phylogenetic analysis [17]. The G protein is one of the main targets associated with induction of protective immunity [18] and the high variability of the protein could indicate that the acquired immune response constitutes an important selective pressure. Previous studies have shown that quasispecies of BRSV are present *in vivo*. The role of this quasispecies distribution is uncertain, but it could represent a reservoir for virus adaptation during infection [19,20]. Antigen variation of the G protein as an escape mechanism in relation to the use of vaccines have been discussed [4,21]. Vaccines against BRSV are rarely used in Norway.

There is only one serotype of BRSV [22]. Four antigenic subtypes called A, B, AB and untyped have been defined based on MAbs directed against the G protein [23,24]. Nucleotide sequence variation of the G gene divides BRSV into six different genetic subgroups, while similar analysis of the N and F genes only result in five subgroups, illustrating the higher rate of evolution of the G gene. Isolates of BRSV from Northern Europe, including Denmark and Sweden, cluster in subgroup II [17].

The aims of the study were to investigate the etiological role of BRSV and other respiratory viruses in epidemics of BRD and to perform phylogenetic analysis of Norwegian BRSV strains.

## Methods

### Selection of herds

Included in the study were 21 cattle herds with acute outbreak of BRD of which five were fattening and 16 were dairy herds. The herds were located in the South-Eastern and Western part of Norway, and samples were collected between March 2009 and June 2011.

A network of veterinary practitioners was established to facilitate early warning of outbreaks. The inclusion criteria for herds were: 1) Acute outbreak of respiratory disease affecting a large proportion of the animals in the herd. 2) The outbreak was assessed to be in an early phase.

### Selection of individual animals

The inclusion criteria for animals were to be in an early stage of infection, i.e. without clinical signs but housed in a pen with diseased animals or having mild coughing and fever. On average five animals was included from each herd, but due to practical limitations, i.e. available animals, the number of animals ranged from two to seven. A total of 104 animals were sampled. In one herd (#17), an additional 93 animals were included. These had coincidently been sampled in the period from six days before, until the day of the outbreak in connection with another research project, and they did not show clinical signs of respiratory disease at the time of sampling.

### Sampling and data collection

From 17 herds sampling was conducted by the same veterinary surgeon and samples were transported to the laboratory the same day. Both serum samples and nasal swabs from 86 of the animals in these herds were collected. In 16 of these herds transtracheal aspirate (TA) or broncheoalveolar lavage (BAL) were collected from 65 of the same animals. In the last one, only serum and nasal swabs were collected because of difficulties in collecting BAL samples due to the large size of the animals. Due to practical reasons, the sampling of TA was performed instead of BAL in the first seven herds included in the study. From the 93 animals sampled before the outbreak in herd #17, only nasal swabs were collected. From additional four herds sampling was conducted by veterinary practitioners and was limited to nasal swabs and sera. These samples were sent by mail to the laboratory and were received the following day.

Paired serum samples were collected from 94 animals of the 21 herds. The samples were collected with three to five weeks interval. Ten animals from five herds were not available for the second sampling (dead/slaughtered or sold). The TA or BAL samples were collected at the same day as the collection of the nasal swabs and the first serum sample. Table 1 shows an overview of animals tested and the laboratory methods used.

**Table 1:** Overview over analysed material from animals in herds with acute outbreak of respiratory disease

**Sample category**	**No. of herds sampled**	**No. of animals analysed**	**Method of detection**
Paired serum	20	94	ELISA
Single serum	1	10	ELISA
Nasal swabs*	21	86	RTqPCR
BAL*	9	37	RTqPCR
TA*	7	19	RTqPCR

The ELISA detected antibodies against BRSV, BCoV and BPIV3, and the RTqPCR detected RNA of BRSV.

*BAL* broncheoalveolar lavage, *TA* tracheal aspirate. Nasal swabs* and TA* or BAL* from herds that were negative based on serology were not analysed.

The BAL was obtained by instilling 100 ml Hanks balanced salt solution (HBSS) via the nostrils into the bronchia through a silicone rubber tube. The diameter of the tube was 10 mm with a 2.5 mm inner channel. Approximately 30 ml was collected. The TA was performed by instilling 40 ml HBSS via a polythene tube. The tube had a diameter of 1.57 mm and an inner channel of 1.14 mm and was passed through an incision in the skin and trachea through a catheter with a diameter of 2.7 mm placed in the trachea. Prior to the incision in the skin, five ml of local anaesthetics containing 100 mg of lidocaine and 180 μg adrenaline was administered subcutaneously around the incision site. Sedation of the animals was performed prior to both the BAL and TA by an intramuscular injection of 0.1 mg/kg of xylazine.

The study was based on natural outbreaks of BRD. The samples taken and procedures performed for diagnostic purposes were according to standard veterinary practice. Therefore it was concluded that formal ethics approval was not necessary. This is in line with the Norwegian Regulation on Animal Experimentation, where it is exemption for treatment and surgery conducted as part of a clinical veterinary procedure, when a recognized method is used.

### Antibody detection

Sera were stored at -20°C until they were analysed. An indirect ELISA that detects antibodies to BRSV, bovine parainfluenza virus 3 (BPIV3) and bovine coronavirus (BCoV) (SVANOVIR® BRSV-Ab, PIV3-Ab and BCV-Ab, Svanova Biotech AB, Uppsala, Sweden) was used following the manufacturer’s instructions. In brief, the reading of the optical density (OD) at 450 nm was corrected by the subtraction of OD for the negative control antigen, and percent positivity (PP) was calculated as (corrected OD/positive control corrected OD) × 100. The sensitivity and specificity of the tests reported by the manufacturer was 94.6% and 100% for BRSV, 95.4% and 98.5% for BPIV3 and 100% and 84.6% for BCoV respectively. The animals were regarded as acutely infected with BRSV, BCoV and/or BPIV3 if the first sample was negative and the second sample was positive, or if the PP increased at least 70%.

The difference in mean herd size of the herds with only one viral agent detected by serology versus those with several viral agents was analysed with a One-way analysis of variance.

### Real time PCR (RTqPCR)

Animals from herds found to have an acute BRSV infection as based on the serologic results, or with ambiguous results, were analysed by RTqPCR. Nasal swabs and lavages were analysed (Table 1). From 36 animals both nasal swabs and BAL were analysed.

The nasal swabs and 0.3 ml of the lavages were put directly in 0.5 and 1.5 ml of RNAlater® (Life Technologies™, Carlsbad CA) respectively, and stored at 4°C for 24 h and then at -80°C until analysis. Samples were thawed and centrifuged at 4000 × g for 7.5 min. The supernatant was removed, and the remaining pellet was dissolved in 300 μl of RNAse free H_2_O. RNA was extracted from 150 μl of this dissolution using QIAamp® Viral RNA (Qiagen) according to the manufacturer’s instructions, resulting in RNA diluted in 60 μl buffer. Detection of BRSV in nasal swabs and BAL samples was performed by using five μl of the RNA solution in a BRSV specific RTqPCR (TaqVet™, Laboratoire Service International, Lissieu, France) amplifying fragments of the BRSV N gene, in Stratagene Mx3005p real-time thermal cycler (Agilent Technologies, Santa Clara, CA). All samples were tested in duplicates. Negative controls were included in the RNA extraction and the RTqPCR. A sample was regarded positive for BRSV if the cycle threshold (Ct) was below 45 for both parallels following the manufacturer’s recommendation.

### Sequencing of G gene

A nested PCR was used to amplify a 541 nucleotide fragment of the BRSV G gene using primer sets as previously described [17]. In brief, RNA was incubated at 65°C for five min and chilled on ice, and RT reaction was run using Superscript III RT (Invitrogen) and the G2.5 primer and incubated at 55°C for 60 min. For the nested PCR the primer sets G2.5, F2.7 and VG1, VG4, respectively were used [17]. The cycling conditions were 40 cycles with 95°C for 20 s, 55°C for 20 s and 72°C for 30 s using the PfuUltra II Fusion HS (Agilent Technologies) DNA polymerase. The cycles were preceded by a denaturation step at 95°C for one min and completed by an elongation step at 72°C for three min. The PCR products were separated by agarose gel electrophoresis, purified using the QIAquick PCR Purification protocol (Qiagen), and nucleotide sequences were obtained (Sanger sequencing) from a total of 24 samples (GATC Biotech AG, Germany). Primer sequences were removed prior to analysis. The sequences of animals from 10 herds and from a Norwegian BRSV isolate from 1976 (A1/N/76), which also originated from an epidemic in the South-Eastern area of Norway, were included. The nucleotide sequences used in this study have been submitted to GenBank and assigned the accession numbers KF501149 – KF501172.

### Phylogenetic and statistical analysis

Contigs of the sequences were made using Vector NTI software package (Invitrogen), and nucleotide sequence alignments were performed using Mega 5 [25] with the Maximum Likelihood method based on the JTT matrix-based model. The pairwise nucleotide distances were estimated using the Tamura-Nei model ignoring positions with gaps. Initial trees for the heuristic search were obtained automatically; when the number of common sites was < 100 or less than one-fourth of the total number of sites, the maximum parsimony method was used. Otherwise BIONJ method with MCL distance matrix was used. Bootstrap values of 1000 replicates were used to assess the nodal support.

JMP 9 (SAS Institute Inc., Cary North Carolina, USA) was used for performance of the One-way analysis of variance.

## Results

### Serology

The ELISA results are presented in Table 2. Of the individuals animals 64 out of 94 (68%) were found to have an acute BRSV infection based on antibody response. Fifteen out of the 94 (16%) were found to be positive for BCoV, and for BPIV3 the number was 11 out of 94 (12%). On herd level, 16 (80%), seven (35%) and three (15%) were found to have animals with antibody response to BRSV, BCoV and BPIV3, respectively. Antibody response only against BRSV was detected in 11 herds, only against BCoV in two herds and only against BPIV3 in one. Antibody responses against both BRSV and BCoV were detected in two herds, while in three herds antibody response against all three viruses were found. In one of the two remaining herds, the animals were seronegative or had a decrease in antibody titre for all three viruses. In the last herd, antibodies against all three viruses were detected, but only single serum samples were available. Concequently, the serological results of this herd were inconclusive and excluded from the calculations based on the serological results.

**Table 2:** Serological results from animals in herds with outbreak of respiratory disease

**Herd size**	**Herd no.**	**Mean age of sampled animals**	**BRSV infection No. pos/sampled**	**BPIV3 infection No. pos/sampled**	**BCoV infection No. pos/sampled**	**Serological detection**
13	20	3.9 yrs	5/5	0/5	0/5	BRSV
20	1	2.3 yrs	1/2	0/2	0/2	BRSV
24	21	3.5 yrs	3/3	0/3	0/3	BRSV
27	16	3.0 mo	4/5	0/5	0/5	BRSV
51	6	2.2 mo	1/5	0/5	0/5	BRSV
55	4	1.2 yrs	7/7	0/7	0/7	BRSV
71	7	1.2 yrs	6/7	0/7	0/7	BRSV
74	3	1.7 mo	0/4	0/4	4/4	BCoV
83	11	7.3 mo	3/5	0/5	0/5	BRSV
95	14	3.8 mo	1/3	0/3	0/3	BRSV
136	13	4.8 mo	3/3	0/3	1/3	BRSV/BCoV
171	8	10.5 mo	6/6	0/6	0/6	BRSV
193	18	7.1 mo	4/5	4/5	2/5	BRSV/BPIV3/BCoV
210	10	5.5 mo	5/5	0/5	0/5	BRSV
210	15	3.7 mo	5/5	0/5	1/5	BRSV/BCoV
220	19	2.7 mo	0/5	0/5	1/5	BCoV
250	12	5.7 mo	0/3	2/3	0/3	BPIV3
256	5	1.4 mo	0/5	0/5	0/5	-
265	17	4.7 mo	5/6	2/6	5/6	BRSV/BPIV3/BCoV
300	9	1.6 yrs	5/5	3/5	1/5	BRSV/BPIV3/BCoV
**Total**			**64/94**	**11/94**	**15/94**	

The herds are listed according to size, i. e. the number of animals in the herds at first sampling. The ratio of animals with acute antibody response against BRSV, BPIV3 and/or BCoV out of investigated animals is shown.

The results are based on detection of antibodies in paired serum samples. Animals were defined as positive for infection if they seroconverted or if the antibody titre increased ≥ 70%. The mean age is at first sampling.

The mean size of the herds with co-infections was significantly larger (p = 0.013) than for the herds with single agent infection. In the herds with co-infections the number of BRSV positive animals dominated (Table 2).

In two of the herds (#2 and 3), the serological results were ambiguous. In herd #2, paired serum samples were lacking. In herd #3 the animals had an increased antibody response against BCoV but in addition one animal had a increase of the level of antibodies against BRSV and BPIV3, but below the threshold set as positive. Samples from the animals with antibody response to BRSV or ambiguous results were analysed for detection of BRSV with RTqPCR.

### Detection of BRSV by RTqPCR

Of 95 collected nasal swabs of the 21 herds, 86 were selected for analysis based on the serological results. Forty-two (49%) of these were positive for BRSV by RTqPCR. Of the 36 animals where both nasal swabs and BAL samples were tested, 20 (56%) were BRSV positive on nasal swabs and 24 (67%) on BAL samples. The difference between the results of the BAL and nasal swabs of these 36 animals was not statistically significant (p = 0.469). Eighteen herds were positive in TA or BAL and nasal swabs by RTqPCR, six animals were positive only for the BAL samples, and two animals only positive for nasal swabs. Of the 19 TA samples analysed, eight were RTqPCR positive. A total of 51 animals from 16 herds were RTqPCR positive for BRSV while two additional herds were positive by ELISA. From the nasal swabs sampled prior to the outbreak in herd #17, four (4.3%) of the 93 were found positive for BRSV.

### Phylogenetic analysis

Nucleotide sequences from the BRSV G protein gene were successfully obtained from 10 herds and from the isolate from 1976. From the additional six herds that were positive for BRSV by RTqPCR, the virus load was below the threshold for achievable sequencing (Table 3).

**Table 3:** Results of RTqPCR for BRSV at herd level with corresponding strain and sequence ID of positive herds

**Herd no.**	**BRSV RTqPCR**	**Retrieved sequence**	**BRSV strain**	**Sequence ID**
1	-	nt		
2	+	+	B	A2/N/09
3	nt	nt		
4	+	+	B	A3/N/09
5	nt	nt		
6	+	+	B	O1/N/09
7	+	-		
8	+	-		
9	-	nt		
10	+	-		
11	+	+	A	O2/N/10
12	-	nt		
13	+	+	A	Os1/N/10
14	+	+	A	O3/N/10
15	+	-		
16	+	-		
17*	+	+	B	O4-4B/N/11
				O4-4S/N/11
18	+	+	B	O5/N/11
19	+	+	A	H1/N/11
20	+	+	A	R1/N/11
21	+	nt		
**Total no. of pos**	**16**	**10**		

*nt* not tested. + = positive and – = negative for RTqPCR detection of virus. *ID for the two different sequences, obtained from the broncheoalveolar lavage and nasal swab of the same animal, are shown.

**Figure 1:**
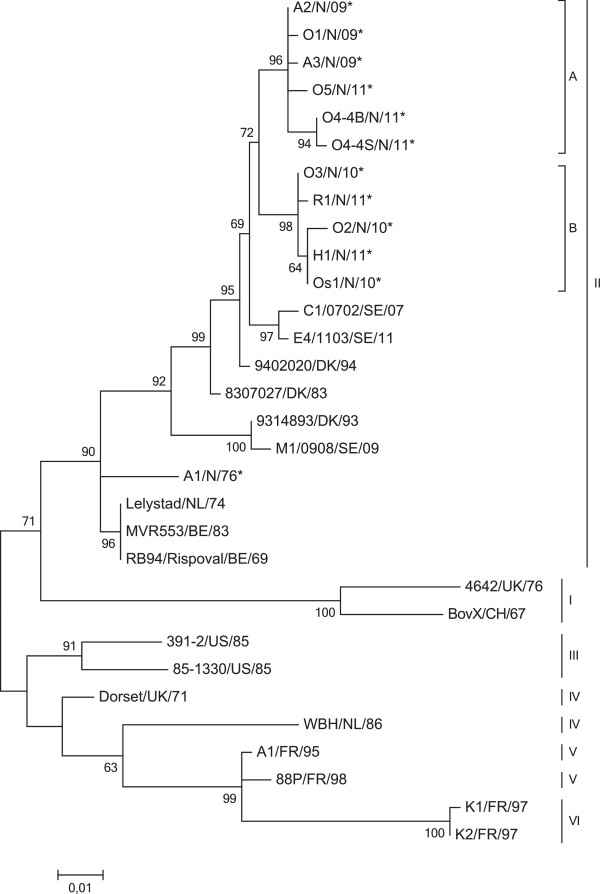
**Phylogeny of the BRSV G gene.** Phylogeny showing the relationship among 31 selected nucleotide sequences based on a 472 nt part from nucleotide number 178 to 649 of the G gene of BRSV. The sequences from this study, labelled with “*”, originated from 10 different farms with outbreaks of respiratory disease in Norway between 2009 and 2011, and one isolate from an outbreak in 1976. Included are also representatives of the six genetic subgroups of BRSV and recent published Swedish and Danish strains. The genetic subgroups (I-VI) and the strain type of the Norwegian sequences **(A or B)** are indicated on the right. The strains are indicated by the isolate name with GenBank Accession numbers AF188585, AF188586, AF188588, AF188604, JN619431, JN619439, JN619446, L27802, M58307, Y08717, Y08718, U92098, U92102, U92103, U24714-U24716, U33539 and U57823. The GenBank accession numbers of the Norwegian strains are: KF501149-KF501155, KF501159, KF501160 and KF501170-KF501172. The bootstrap values are shown next to the branches. The trees are drawn to scale, with branch lengths measured in the number of substitutions per site.

The Norwegian BRSV nucleotide sequences obtained from 2009–2011 all belonged to subgroup II along with other Scandinavian isolates (Figure 1). The total nucleotide difference of the gene fragment was only between 0.15% and 0.31%. The isolate from 1976 (A1/N/76) formed a separate branch (Figure 1). The difference between A1/N/76 from the sequences from 2009–11 samples was between 4.9% and 6.2%.

**Figure 2:**
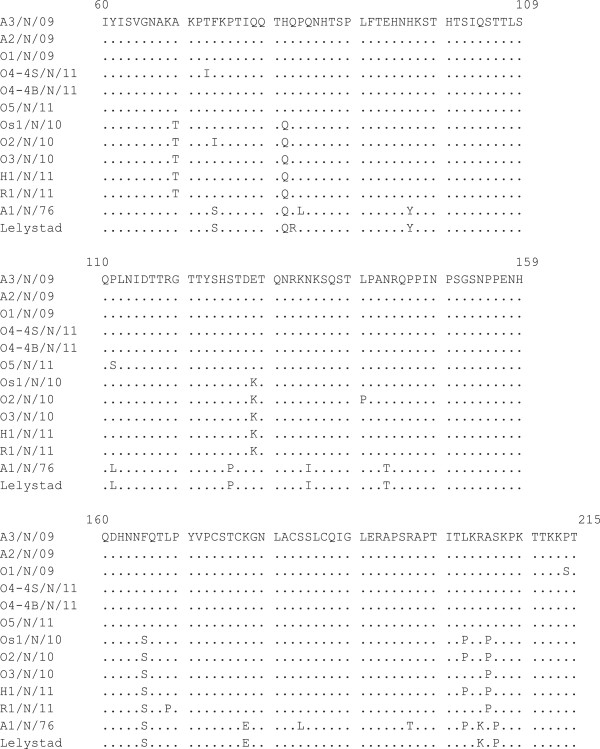
**Amino acid sequences 60 to 215 of the G protein gene of BRSV isolates.** Comparison of deduced amino acid sequences. Designations on the left indicate the isolate ID. The GenBank accession number of the Lelystad strain is U33539.

The deduced amino acids from the Norwegian sequences separated in two distinct strains called A and B. The occurrence of the A and B strains overlapped both temporarily and geographically. Six nucleotides and five amino acids systematically differed between strain A and B (Figure 2) i.e. five of the six consistently differing nucleotides gave nonsynonymous substitutions.

The G protein gene nucleotide sequences obtained from nasal swabs and BAL samples from five of the same individuals in herd #17 were identical, apart from one nucleotide difference found in one sequence obtained from a nasal swab of one animal. This resulted in a substitution from Threonine to Isoleucine. Apart from this single substitution, samples from all animals of herd #17 were identical, including the three sequences obtained from samples prior to disease outbreak in herd #17.

Figures 1 and 2 are deposited in Dryad and can be found at http://doi.org/10.5061/dryad.6k8b1.

## Discussion

The herds that were included in this study represented commercial cattle farms where veterinary practitioners had reported outbreaks of acute respiratory disease. Our results indicated that BRSV is the dominating etiological agent in acute outbreaks of BRD in Norway. This was supported by both serology by ELISA and virus detection by RTqPCR.

In the majority of outbreaks BRSV was the only virus detected. When several agents were detected in the same herd, BRSV was always present and the number of BRSV positive animals was higher than for BPIV3 and BCoV, supporting the view of BRSV as the main pathogen. The herds with two or three respiratory viruses present at the same time were significantly larger than the others. This indicates that the infection dynamics may be more complex in larger herds.

The nucleotide sequences of the BRSV G protein gene from BAL samples and nasal swabs, i.e. from lower and upper respiratory tract, were identical for each individual in herd #17 with the exception of one sequence that had a single substitution of a nucleotide. Similarly, the sequences obtained from this herd collected before and during the disease outbreak were also identical, indicating the dominance of a single virus strain during the outbreak.

This herd is large with extensive purchase of livestock from all parts of the country and is thus at a higher risk of the introduction of different strains of virus.

Only a minor part of the BRSV genome was sequenced, but it is the part where most variation has been found [17,26]. This indicated that during a disease outbreak a virus with identical G genes dominates within a herd or an animal, suggesting that BRSV outbreaks are initiated by virus of apparently high fitness. This is in accordance with previous findings of Larsen et al. [27] that revealed that cell-culture adaption and passages in cells and/or calves did not induce extensive changes in the G protein gene. When the PCR products were generated for sequencing in the present study, a proof-reading polymerase was used to minimise procedure generated mutations. The sequences were obtained directly from PCR products and thus mirror the consensus base at each position, i.e. the dominating sequence. However, low abundant sequence variants would not have been detected by this procedure.

There was no statistical significant difference in the RTqPCR results of the BAL or nasal swabs from the same animal. Although the results are based on samples from a limited number of animals they indicate that nasal swabs as samples for RTqPCR testing can be useful. This is important knowledge for the collection of samples from the field, as the nasal swabs are much more convenient to collect than BAL.

All the BRSV G protein sequences obtained in this study, including the A1/N/76 isolate, belonged to genetic subgroup II along with the other most recent North European isolates [17]. The deduced amino acid sequences retrieved in our study could be divided into two clades where six nucleotide substitutions systematically gave a difference of five amino acids between the clades. Prozzi et al. [28] showed that the antigenic subgroups are related to the sequence variability of the central conserved region (codon position 154 to 192) of the G protein. In another study, Langedijk et al. [29] demonstrated that two point mutations at positions 180 and 205 defined the classification of the antigenic subgroups. Some of the differences observed in the present study were located in the area that is determinative for antigen subgrouping [29] as well as in the conserved central hydrophobic region [17]. Amino acid residue 205 of the Norwegian strain B is a Pro, while for strain A and the other aligned isolates Ala is present. Proline can impede the protein folding and thus potentially modify properties of the G protein. In the study of the evolution of BRSV by Valarcher et al. [17], Ala_205_ was found for subgroups I and II while Thr_205_ was present for the other subgroups. It was speculated whether this could introduce an O-glycosylation site that could modify the antigenicity of the linear epitope described for positions 199 to 209 [29]. Furthermore the Ala_205_ to Thr_205_ substitution caused lack of binding of a specific MAb used for antigenic subgrouping of BRSV [29]. This illustrates the central role of residue 205 and could indicate that the Ala_205_ to Pro_205_ mutation found in our material could be a factor that would ease the similar spatiotemporal circulation of both strains. It is interesting that the two distinct strains were not found within the same herd. A recent Swedish study with samples from different parts of Sweden showed that the viral G protein sequences were highly similar [30] and could not be divided into distinct strains while in Denmark extensive sequence divergence (11%) of the G protein gene was found in recurrent outbreaks in closed herds [19].

Based on the phylogenetic analysis, the Norwegian BRSV strains showed the highest degree of homology with recently published Swedish [30] and Danish strains [19], supporting the theory of a geographical and temporal clustering. The A1/N/76 isolate from 1976 formed a separate branch in subgroup II, clearly different from the current Scandinavian sequences. The distant relation between this isolate and the current Norwegian strains do not indicate if the current strains are direct descendants, or if they only originate from a common ancestor. This isolate, causing a large outbreak of BRSV in Norway at that time, was originally thought to be introduced to Norway by an animal imported from Denmark [12]. Further studies, including samples from the time period between these isolates, are needed to determine the origin of the current strains.

## Conclusions

The present study shows that BRSV is currently the most important etiological agent in epidemics of respiratory disease in cattle in Norway, and that it often acts as the only viral agent. The current Norwegian strains of BRSV belong to the genetic subgroup II along with other North European isolates, supporting the theory of geographical and temporal clustering of BRSV. The currently circulating BRSV could be divided into two strains that were present in the same geographical area at the same time.

## Competing interest

The authors declare that they have no competing interests.

## Authors’ contributions

TBK participated in the study design and collection of samples, conducted all laboratory and statistical analyses, participated in interpretation of analyses, and drafted the manuscript. ER participated in the study design, provided oversight of laboratory analysis, and participated in interpretation of analyses and helped to draft and critically revise the manuscript. MS participated in study design and participated with interpretation of analyses and helped to draft and critically revise the manuscript. All authors have read and approved the final manuscript.
